# Determination of Total Silicon and SiO_2_ Particles Using an ICP-MS Based Analytical Platform for Toxicokinetic Studies of Synthetic Amorphous Silica

**DOI:** 10.3390/nano10050888

**Published:** 2020-05-06

**Authors:** Federica Aureli, Maria Ciprotti, Marilena D’Amato, Emanueli do Nascimento da Silva, Stefano Nisi, Daniele Passeri, Angela Sorbo, Andrea Raggi, Marco Rossi, Francesco Cubadda

**Affiliations:** 1 Istituto Superiore di Sanità-National Institute of Health, 00161 Rome, Italy; federica.aureli@iss.it (F.A.); maria.ciprotti@iss.it (M.C.); marilena.damato@iss.it (M.D.); angela.sorbo@iss.it (A.S.); andrea.raggi@iss.it (A.R.); 2 Department of Chemistry, Institute of Exact and Biological Sciences, Federal University of Ouro Preto, Ouro Preto 35400000, MG, Brazil; emanueli.silva@ufop.edu.br; 3 Institute of Chemistry, University of Campinas, Campinas 13083970, SP, Brazil; 4 Gran Sasso National Laboratory, National Institute of Nuclear Physics (LNGS-INFN), 67100 Assergi (AQ), Italy; stefano.nisi@lngs.infn.it; 5 Department of Basic and Applied Sciences for Engineering, University of Rome Sapienza, 00161 Rome, Italy; daniele.passeri@uniroma1.it (D.P.); marco.rossi@uniroma1.it (M.R.); 6 Research Center for Nanotechnology Applied to Engineering of Sapienza University of Rome (CNIS), University of Rome Sapienza, 00185 Rome, Italy

**Keywords:** synthetic amorphous silica, tissue levels, ADME, microwave digestion, silicon determination, inductively coupled plasma mass spectrometry, polyatomic interferences, analytical quality control, single particle ICP-MS

## Abstract

Synthetic amorphous silica (SAS), manufactured in pyrogenic or precipitated form, is a nanomaterial with a widespread use as food additive (E 551). Oral exposure to SAS results from its use in food and dietary supplements, pharmaceuticals and toothpaste. Recent evidence suggests that oral exposure to SAS may pose health risks and highlights the need to address the toxic potential of SAS as affected by the physicochemical characteristics of the different forms of SAS. For this aim, investigating SAS toxicokinetics is of crucial importance and an analytical strategy for such an undertaking is presented. The minimization of silicon background in tissues, control of contamination (including silicon release from equipment), high-throughput sample treatment, elimination of spectral interferences affecting inductively coupled plasma mass spectrometry (ICP-MS) silicon detection, and development of analytical quality control tools are the cornerstones of this strategy. A validated method combining sample digestion with silicon determination by reaction cell ICP-MS is presented. Silica particles are converted to soluble silicon by microwave dissolution with mixtures of HNO_3_, H_2_O_2_ and hydrofluoric acid (HF), whereas interference-free ICP-MS detection of total silicon is achieved by ion-molecule chemistry with limits of detection (LoDs) in the range 0.2–0.5 µg Si g^−1^ for most tissues. Deposition of particulate SiO_2_ in tissues is assessed by single particle ICP-MS.

## 1. Introduction

Synthetic amorphous silica (SAS) has been used in the food industry sector as food additive, with the E number E 551, for decades. Food-grade SAS is used for a variety of functions, e.g., as an anticaking agent, antifoaming agent or flow aid in powdered food, but also in food processing as a clarifying/fining agent in the juice, oil and brewery sector or as flavor carrier [1]. For instance, in the EU, E 551 is an authorized food additive in 22 categories of food and food supplements (in solid or liquid form), as well as in a number of food-grade components (additives, enzymes, flavorings, nutrient sources) at levels ranging from 2000 to 30,000 mg/kg or quantum satis [2]. In addition to its use as a food additive, E 551 is also used in cosmetics (notably as an abrasion additive in toothpastes) and in pharmaceuticals (as a free-flow additive, carrier, retardant agent and tableting aid) [3].

E 551 is a nanomaterial produced synthetically by either a vapor-phase process yielding pyrogenic (or fumed) SAS, or by a wet process yielding precipitated silica or silica gel. These production processes lead to the formation of small primary SiO_2_ nanoparticles (mostly <20 nm), which may form agglomerates and/or aggregates depending on the conditions of production and use; the nanosized nature of E 551 has been unambiguously documented by a large number of studies [1,4,5,6,7,8,9,10,11]. Owing to the widespread use, average dietary exposure of the general population to SAS is estimated in the range of 2–25 mg/kg bw per day (‘brand-loyal scenario’, with high-level exposure in the range 4–61 mg/kg bw per day) [2], to which non-dietary oral exposure (via drugs and toothpaste) has to be added.

A recent review highlighted that toxicity of silica nanoparticles in vitro is size, dose, and cell type dependent but the correlation between in vitro and in vivo toxicity remains less well established mainly due to improper—unrealistically high—dosing both in vitro and in vivo [12]. As a matter of fact, two very recent in vivo studies focusing on longer term exposure (3–18 months) at doses in the expected range of dietary intake did find adverse effects in the liver, kidney and thyroid [13,14,15]. The findings of these studies are consistent with the conclusions of a previous nano-specific risk assessment, which highlighted that SAS is a biopersistent material prone to accumulation in tissues upon long-term exposure with daily consumption and SAS in food may pose a health risk [16]. van Kesteren et al. [16] emphasized the importance of kinetics, more specifically tissue distribution and elimination, in addressing the toxic potential of SAS and understanding how it may be affected by the physicochemical characteristics of the different forms and types of SAS. The crucial importance of toxicokinetic information is pointed out by the EFSA guidance on risk assessment of nanomaterials in food, which highlights that size-related properties, shape or surface characteristics of the particles can affect the ADME (absorption, distribution, metabolism, and excretion) behavior [17].

ADME studies for nanomaterials are extremely challenging. An appropriate measurement system must be available for detecting the nanomaterial in organs, tissues and other biological samples. Labelling of the nanomaterial may be used, either directly (radioactive or stable isotopes) or indirectly (fluorescent dyes or radiolabels). Limitations of labelling lie in the difficulty to apply such an approach to real-world materials (i.e., label the particles as synthesized via industrial processes), non general applicability (e.g., isotopic labels), the risk that the label detaches from the particles (especially for fluorescence labelling). Some labelling systems may also modify the properties of the nanomaterials [17]. An attractive alternative is detecting the nanomaterial via its chemical composition. For inorganic nanomaterials, the elemental composition can be tracked effectively via inductively coupled plasma mass spectrometry (ICP-MS), a technique that offers the detection power needed for measuring even low amounts of particles via the concentration of their characteristic chemical element. In addition ICP-MS has the potential to directly detect particles—when used in single particle mode (‘single particle ICP-MS’) [18] or coupled with asymmetric flow field flow fractionation [19]—offering at the same time chemical specificity, a feature that is rarely available in sizing techniques.

In the case of nanosized silica, performing toxicokinetic studies by measuring silicon via ICP-MS detection presents several challenges. The most critical ones are contamination (due to the ubiquitous presence of silicon in reagents and of silica in labware and analytical equipment), the existence of a substantial biological background (silicon is naturally-occurring in tissues), the need to convert (chemically resistant) silica particles into soluble silicon, the severe spectral interferences affecting ICP-MS detection of silicon, and the lack of reference materials with a biological matrix for checking the accuracy of measurement. The analytical strategy presented herein addresses all these challenges.

## 2. Materials and Methods

### 2.1. Standards and Reagents

A Milli-Q Element System (Millipore, Molsheim, France) was used to obtain high purity water for sample preparation and all dilutions. Nitric acid (HNO_3_, Carlo Erba Reagenti, Rodano, Italy) and hydrofluoric acid (HF, Carlo Erba Reagenti, Rodano, Italy) were of ultrapure grade. Calibrants and the internal standard (germanium) for total silicon (Si) measurements were prepared from certified solutions of 1 mg mL^−1^ (High Purity Standard, Charleston, SC, USA) by dilution with 0.5% v/v HNO_3_. Two commercial SAS materials used in the food industry were provided in prescored glass ampoules by the Joint Research Center repository of the European Commission (Ispra, Italy) in the frame of the Nanogenotox European Joint Action [20]. These representative test materials consisted of uncoated amorphous hydrophilic silica obtained by different production systems, i.e., precipitated (NM-200) and thermal (NM-203); a comprehensive characterization is available elsewhere [10,21]. For clarity, some of the main physicochemical features are summarized in the supplementary material (Table S1).

The two SAS materials were used in method development and validation along with the QC-ISS bovine liver-based quality control material (QCM) produced in-house (see Section 2.4), the certified reference material SRM 1548a Typical Diet (NIST, Gaithersburg, MD, USA), and the accuracy control material Seronorm whole blood L-1 lot. 1003191 (SERO, Billingstad, Norway); the two latter have information values for total Si of 78.7 µg Si g^−1^ and 0.81 µg Si g^−1^, respectively.

### 2.2. Instrumentation

An Elan DRC II ICP mass spectrometer (Perkin Elmer, Norwalk, CT, USA) was used as Si specific detector. To minimize instrumental Si background, the standard sample introduction system of the ICP mass spectrometer was replaced with inert (non-quartz) components, namely a ceramic D-torch and a sapphire injector (Perkin Elmer, Norwalk, CT, USA), used with a PFA cyclonic spray chamber and concentric nebulizer (Elemental Scientific Inc., Omaha, NE, USA).

Methane, ammonia, hydrogen (10% in Ar) and oxygen (all 99.995%, Gruppo Sapio, Monza, Italy) were investigated as reaction gases to minimize the polyatomic interferences affecting Si isotopes. The optimized ICP-MS operating parameters are summarized in Table 1.

### 2.3. Sample Preparation

#### 2.3.1. Decontamination Procedures

The use of glass equipment was avoided during sample preparation to prevent Si contamination. Disposable polypropylene tubes were filled up with HNO_3_ 2 M and HF 1 M v/v, left to stand overnight and then rinsed three times with Milli-Q water. To prevent carryover, after each sample digestion Teflon vessel were submitted to two cleaning microwave irradiation cycles with a 10 mL-mixture containing 5 mL HNO_3_ (approx. 13 M), 1 mL HF (approx. 4 M) and 4 mL H_2_O v/v.

#### 2.3.2. Sample Collection

The analytical approach described in this paper was developed for the SAS toxicokinetic study performed within the Nanogenotox Joint Action; rat tissues and rat blood from that study were used for analytical method development. Briefly, suspensions of the two materials were prepared at a concentration of 6 mg mL^−1^ in sterile normal saline (NaCl 0.9% w/v) by probe sonication on ice for 16 min at 10% amplitude using a Sonopuls ultrasonic Homogenizer HD3200 series (Bandelin electronic GmbH, Berlin, Germany) equipped with a SH 213 G booster horn and a sterile KE 76 tapered tip. The two SAS materials were administered via gavage or intravenously at a dose of 20 mg/kg bw/d for one (IV route) and five consecutive days (IV and oral route), followed by a recovery period of 14 (oral route) or 90 days (IV route). Samples of liver, spleen, kidneys, lungs, hearth, brain, ovaries, testes in IV studies, and GI tract, liver, spleen and mesenteric lymph nodes in the oral study were collected for chemical analysis (*n* = 3/sex/time point for organs; *n* = 2/time point for blood). More details on the toxicokinetic study can be found in Cubadda et al., 2020 [22].

#### 2.3.3. Microwave Digestion for Total Si Determination

All samples manipulations were carried out in clean room conditions under a laminar flow box (Spetec GmbH, Erding, Germany). In order to reduce sample handling and contamination risk entire organs entailing lungs, heart, kidney, ovaries, testis, mesenteric lymph nodes were digested with the exception of the largest organs, namely liver, spleen and gastrointestinal tract that were homogenized beforehand. The GI tract was subsampled for Si determination and submitted to a cleaning procedure consisting in the mechanical removal of digestion residues inside the tract using ultrapure water.

Complete sample dissolution was accomplished by means of microwave irradiation at high temperatures with mixtures of HNO_3_, H_2_O_2_ and small amounts of HF [7,23]. The HF quantity added was selected in order to give a final HF concentration in the digested samples <0.1%. At these concentrations, fluorides precipitation is prevented and boric acid addition to eliminate excess HF is not necessary [24,25].

Largest organs, namely liver, spleen and small intestine, were digested in triplicate by means of a Teflon closed-vessel system equipped with temperature control (Milestone Ethos E Prolab station, FKV, Bergamo). Typical conditions for closed-vessel digestion were 2 g fresh sample treated with 5 mL HNO_3_ (13 M) and 0.025 mL HF (4 M), overnight pre-mineralization, and then addition of 2 mL of H_2_O_2_ before irradiation. The microwave program used was as follows: 10 min ramp to 120 °C; 7 min at 120 °C; 9 min ramp to 190 °C; 15 min at 190 °C (maximum power 1000 W). This digestion method was used also for characterization of the total Si content of NM-200 and NM-203 dispersions; for this purpose, 0.2 g of the suspensions were digested under the same conditions as fresh samples.

An approach for the simultaneous microwave digestion of 60 samples in polypropylene tubes was developed for smaller organs. Lungs, kidneys, brain, heart, testes, ovaries, and mesenteric lymph nodes (pooled) were digested as whole organs in disposable Falcon™ decontaminated tubes by means of microwave irradiation in a Multiprep apparatus (FKV, Bergamo, Italy). Tissue samples were weighed, added with 5 mL HNO_3_ (13 M) and 0.025 mL HF (4 M), and left to stand overnight (predigestion step); the following morning, after addition of 2 mL H_2_O_2_, samples were irradiated using the following program: 1 h ramp to 90 °C; 7 h at 90 °C. Blood samples (1 mL) were digested with a mixture of HNO_3_ (13 M, 2 mL), HF (4 M, 0.015 mL) and H_2_O_2_ (1 mL), and microwave irradiation using a 1 h ramp to 90 °C and 5 h at 90 °C.

### 2.4. Preparation and Characterization of the Quality Control Material (QCM)

The few biological reference materials available to check the quality of Si determination have limitations in terms of the type of matrixes (tissues) covered; in addition they all have indicative and not certified Si concentration values. Therefore, an internal QCM (QC-ISS) was prepared starting from a fresh bovine liver sample as follows. After removing membranes, ligaments, and large blood vessels, the liver sample was thoroughly homogenized in an automatic blender (Buchi, Cornaredo (MI), Italy). The sample was spiked with a known amount of Si, mixed again and then freeze dried by means of a LyoLab 3000 system (Heto-Holten, Alleroed, Denmark). As the final step, the sample was ground by means of an automatic agate pestle mill (Retsch, Haan, Germany), passed through a 125 µm sieve to obtain a fine powder and transferred in pre-scored vials, previously rinsed with HNO_3_ 10%. The resulting samples were stored in a desiccator cabinet and used for method development and included as internal quality control in each analytical batch.

The Si concentration in QC-ISS was characterized by ICP-DRC-MS and two independent complementary techniques, namely ICP-optical emission spectroscopy (ICP-OES) and high resolution ICP-MS (HR-ICP-MS). Analyses were carried out following oxidative digestion in the same conditions adopted for fresh organs. Analytical conditions for the complementary techniques can be found in the Supplementary Materials (Figure S1, Tables S3 and S4).

### 2.5. Method Validation and Analytical Quality Control

The selected microwave digestion approach converted SiO_2_ nanoparticles into soluble Si, which was accurately quantified using chemical resolution of polyatomic interferences in the DRC. The analytical performance of the method developed for Si determination was subjected to a detailed characterization, including limit of detection (LoD), limit of quantification (LoQ), trueness, linearity, repeatability and reproducibility (within-laboratory, i.e., intermediate precision). The method was tested on real samples to ensure it was fit for purpose and validated.

As there are no certified reference materials available for SiO_2_ nanoparticles or total Si in a biological matrix, analytical quality control (QC) was accomplished by the use of matrix-matched quality control samples prepared in house (QC-ISS) and batches of Seronorm Whole Blood, for organs and blood, respectively. SRM 1548a was used as matrix-matched reference material for standard rodent diets. QC samples were included in each analytical batch.

### 2.6. Characterization of the Si Content of Maintenance Diets

Standard rodent diets were characterized for their total Si content. Three different feed suppliers provided five pelleted diets. Samples were ground with an automatic agate pestle mill (Retsch, Haan, Germany), microwave digested following the same conditions used for larger organs and analyzed by ICP-DRC-MS. Results were cross-validated with ICP-OES as a complementary technique.

### 2.7. Determination of SiO_2_ Particles by sp-ICP-MS and SEM-EDX

Liver samples collected from animals at day 90 after single IV NM-203 administration were characterized for the presence of SiO_2_ in particulate form. Livers from control and treated animals were enzymatically digested for extracting the particles [18]. Briefly, samples of liver homogenates were submitted to cup horn (indirect) sonication for 5 min at 20% amplitude using a Sonopuls HD 3200 apparatus equipped with a BB6 cup horn in Tris–HCl buffer 50 mM at pH 8 and 10% sodium dodecyl sulphate. Subsequently, samples were incubated with 2 mg mL^−1^ Proteinase K (Sigma Aldrich, Dorset, UK) at 45 °C for 1 h under mechanical agitation. Before cooling, samples were sonicated at 20% amplitude for 5 min and immediately diluted for analysis. Procedural blanks were extracted and analyzed in parallel. The optimized ICP-DRC-MS conditions of Table 1 were used for single-particle ICP-MS analysis with the following variations: for sample uptake a Perimax 16 peristaltic pump antipulse (Spetec, Erding, Germany) operating at 0.5 mL min^−1^ was used; transient ^28^Si signals were recorded for 60 s with a dwell time of 3 ms. The reference material NIST SRM 8013 Gold nanoparticles (NIST Gaithersburg, MD, USA) was used as calibrant.

Scanning electron microscopy analysis was performed by a CrossBeam Workstation^®^ (FIB-SEM) AURIGA from Carl Zeiss Microscopy (Oberkochen, Germany) equipped with a Quantax energy dispersive X-ray (EDX) system using an XFlash 6 silicon drift detector with a resolution of 121 eV. Electrons at 15 keV by were used to perform elemental analysis through EDX.

### 2.8. Statistics

Statistical analysis of results was performed by the Minitab Statistics Package (ver. 17) developed at the Pennsylvania State University (Minitab, PA, USA).

## 3. Results

### 3.1. Interference-Free Detection of Total Si

**Figure 1:**
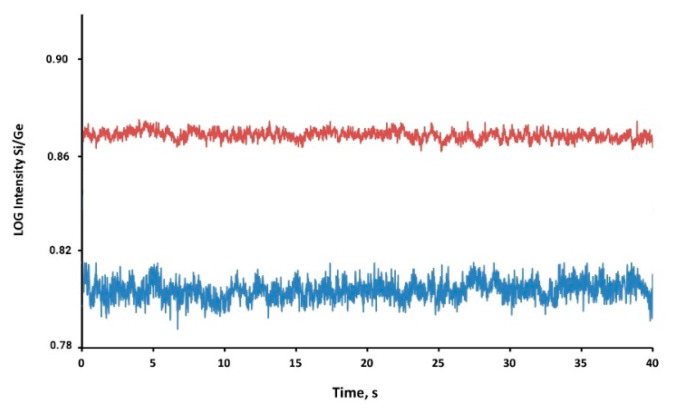
Signal profile of ultrapure water analyzed with the standard configuration in quartz (red line) and after replacement with non-quartz components (blue line). Ratios of logarithms of Si and Ge intensities are shown.

Si determination by quadrupole ICP-MS presents several analytical challenges, the main one being the Si background due to Si release from the quartz sample introduction system of the spectrometer and the spectral interferences on the potentially usable Si analytical masses [4]. Analysis of Milli-Q water showed a systematic decrease of the background Si signal by 20% when quartz was completely removed by the sample introduction system of the ICP-MS (Figure 1).

To remove interferences from ^14^N^14^N^+^ and ^12^C^16^O^+^ at *m/z* 28 and other polyatomic ions on the other Si isotopes (Table S2, Supplementary Materials), thereby improving the ICP-MS LoDs, ion-molecule chemistry was used. Methane, Oxygen, Hydrogen and Ammonia were investigated as reaction gases, and gas flow rate and DRC RPq were optimized for Si detection in a liver matrix. Promising results for the background equivalent concentration (BEC), i.e., the apparent concentration for the background signal based on the sensitivity of the element at the specified mass, were obtained with methane at *m/z* 28. The lower the BEC value, the more easily a signal generated by the element can be discerned from the background [26,27]. Changes in the reaction profiles reflect the different reactivity of ion populations, comprising interfering species and the analyte of interest (Si), and show that reactions of the polyatomic ions take place more efficiently with methane (note the marked decay of the signal intensity and the change of slope for the blank solution in Figure S2, Supplementary Materials). Methane gas flows ≥0.5 units with an RPq in the range 0.50–0.65 were identified as optimal and selected for method characterization and application to real samples.

### 3.2. Characterization of the QCM by a Multi-Technique Approach

The internal reference material prepared as QCM was chemically characterized by means of three different and independent instrumental techniques (ICP-DRC-MS, ICP-OES, HR-ICP-MS). The procedures required to characterize a QCM when an adequate CRM is not available were followed [28,29,30]. Each set of results was checked and confirmed for normality by the Ryan–Joiner test (*p* > 0.05; α = 0.05). The homogeneity of the variances was checked by the Levene’s test at a significance level α = 0.05 and the obtained *p*-value (*p* = 0.534) demonstrated homoscedasticity. Therefore the three datasets were compared through analysis of variance (ANOVA) and the sample means proved to be not statistically different (*p* = 0.791).

Since no significant difference was observed, all data were combined and the overall mean—equal to 20.9 µg Si g^−1^—was used as the target (reference) value for the QCM. The pooled standard deviation—i.e., SD_pooled_ = 1.4 µg Si g^−1^—was taken as an indicator of the material’s standard uncertainty [30].

### 3.3. Method Validation

#### 3.3.1. Limits of Detection and Quantification

LoD and LoQ were determined based on the measurement of twenty independent digestion blanks and calculation of the resulting standard deviation (*s*). The LoD was determined as the analyte concentration corresponding to a 3 *s* value whereas the LoQ was determined as the analyte concentration corresponding to a 10 *s* value. The concentrations in the measurement solution were transformed in tissue concentrations via the applied dilution factor and both limits are expressed as µg Si g^−1^ tissue.

**Figure 2:**
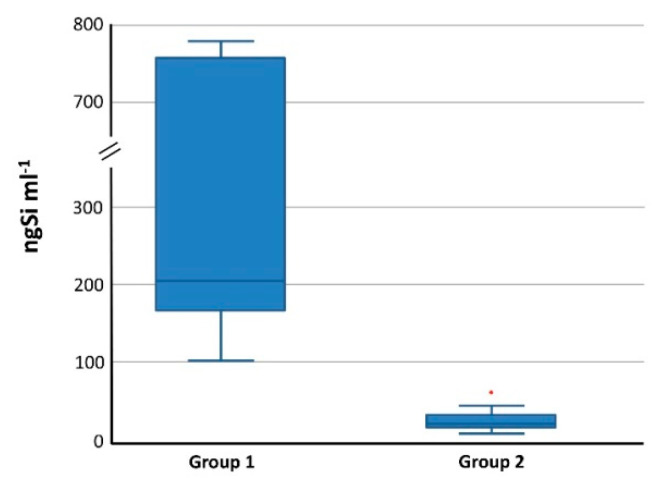
Box plots representing magnitude and variability of background Si concentration in method blanks (digestion reagent mixture after MW irradiation) obtained with standard vessel cleaning (Group 1) and the cleaning procedure used in this study, as described in Section 2.3.1 (Group 2). Outliers shown as red spots are unzoomed for Group 1 (*n* = 2).

If trace concentrations of Si are to be determined, any sample contamination during the whole analytical procedure is a source of bias and results in the increase of the LoD; on the other hand, the LoD can be improved by reducing background noise and ensuring that the contribution from method blanks is low and stable [31,32,33]. When the decontamination procedure described in Section 2.3.1 was applied, Si concentration in digestion blanks decreased by a factor of ~10 (Figure 2) and the variability of Si background associated with labware contamination or carryover effects from previous samples was reduced to negligible levels.

As a result, the LoD and LoQ obtained in this study (Table 2) are between one and two orders of magnitude lower compared to those achieved in other in vivo studies with SiO_2_-based materials [34,35,36,37]. This allowed the administration of low, biologically relevant doses (20 mg/kg bw/d) of the SAS test materials in the oral and the IV toxicokinetic studies for which the analytical method presented herein was developed. Accurate detection of the minute amounts of silicon in blood and tissues resulting from SAS administration of such dose levels was instrumental in gaining a detailed picture of biodistribution and elimination kinetics, determine the relevant parameters and estimate the oral absorption for the precipitated (NM-200) and thermal (NM-203) SAS nanomaterials investigated [22].

#### 3.3.2. Trueness

One of the main focuses of method development in the present study was the complete dissolution and quantitative recovery of SAS from tissues. In the absence of suitable reference material, recovery studies via spiking experiments were used to document conversion of SAS particles into soluble silicon and assess the trueness of analytical results.

Samples of control (i.e., unexposed) animals were pooled, thoroughly homogenized and spiked with known amounts of SAS particle dispersions. The total Si content of the NM dispersions was ascertained by ICP-DRC-MS after microwave digestion (see Section 2.3.3) and measured average values (*x*) matched well with the reference value (*x_ref_*) measured during material characterization [10]. Relative per cent recovery was calculated according to Equation (1) and results are showed in Table 3:

(1) $$R\left(\%\right)=\frac{\bar{x}}{x_{ref}}\times 100$$

SAS was spiked in triplicate at two different levels corresponding to 8 mg Si kg^−1^ and 20 mg Si kg^−1^ to representative target organs, namely liver and spleen. Matrix blanks and spiked samples were submitted to microwave digestion with either HP Teflon Vessel or Multiprep systems and analyzed by ICP-DRC-MS as part of the validation study to assess the net relative per cent recovery according to Equation (2) where ${\bar{x}}_{sp}$ is the measured average value of the spiked samples, ${\bar{x}}^{\prime }$ is the measured average value of the unspiked samples and *x_spike_* is the added concentration:

(2) $$R^{\prime }\left(\%\right)=\frac{{\bar{x}}_{sp}-{\bar{x}}^{\prime }}{x_{spike}}\times 100$$

The acceptance limit was set between 80% and 120%. Average relative recoveries were satisfactory and are summarized in Table 3.

#### 3.3.3. Linearity and Precision

The validity of a linear calibration model was evaluated by generating two six-point calibration matrix-matched curves using standards of soluble silicon in the range of interest, i.e., 25–5000 µg Si L^−1^ for liver, spleen, GI tract and lungs and 25–1000 µg Si L^−1^ for heart, brain, kidneys, testis, ovaries and mesenteric lymph nodes on three different days. The calibration model was accepted if the back-calculated values were within 20% for at least two thirds of the data points [38]. The acceptance criterion was fulfilled since all back-calculated values were between −15% and 12%; therefore a linear unweighted calibration model with a correlation coefficient ≥ 0.999 was adopted.

Measurement repeatability and intermediate precision were evaluated on *n* = 6 samples analyzed in three different days for QC samples (namely, Seronorm Whole blood and QC-ISS) covering different concentration levels. Results expressed as relative standard deviations were 12.4% and 18.8% for Seronorm Whole blood, and 4.8% and 6.3% for QC-ISS, respectively.

Overall, method performance was satisfactory and the method proved to be fit for purpose.

### 3.4. Applications to Real Samples

#### 3.4.1. Maintenance Diet

One of the challenges in undertaking toxicokinetic studies of unlabeled SAS materials (i.e., via Si determination) is the high endogenous Si background in tissues and biological fluids. The Si background concentration depends on the Si amount ingested via the diet, which consists of naturally occurring soluble silicon (orthosilicic acid–OSA–and associated silicon-containing species with high bioavailability) and some polymeric or phytolithic silica in vegetable food (thought to be hydrolyzed to OSA in the GI tract) [39,40,41,42,43]. The elimination half-life of dietary silicon via urinary excretion is in the order of few hours depending on the source [44,45,46,47] highlighting that Si background, at least in fasting blood (and perhaps to a certain extent in some organs), can be limited if animals are acclimated to a non-high Si diet. For this aim, five different standard rat diets were analyzed for their Si content.

**Figure 3:**
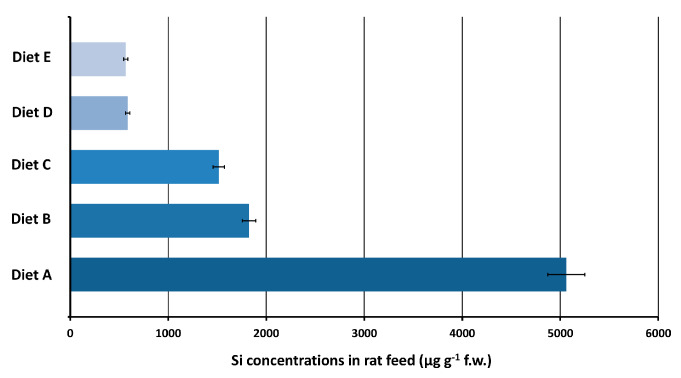
Si concentration range of rodent diets tested in this study.

For quality control, SRM 1548a Typical Diet was analyzed and the found Si value was 82% of the reference (indicative) value. Results (Figure 3) were cross-validated with an independent technique, namely ICP-OES, which gave statistically indistinguishable results.

The results, shown in Figure 3, highlight the wide variability (10-fold) of Si content in rat diets. Diet E, which contained the lowest amount of Si, was selected for use in the SAS toxicokinetic studies to limit the Si background in the blood of experimental animals [22]. As a result, Si background in circulating blood was reduced below the analytical LoD. In addition, Si background in rat organs was found to be below the analytical LoQ in most cases.

#### 3.4.2. Total Si in Blood and Organs

The peak Si concentrations determined by ICP-DRC-MS in rat blood were 5.6 ± 0.4 and 92.0 ± 4.1 µg Si g^−1^ for single and repeated IV administration, respectively [22]. The range of Si concentrations measured in rat tissues are summarized in Table 4 and Table 5 for single and repeated IV administration, respectively [22]. Results showed that liver and spleen are the target tissues for deposition, with Si concentrations ranging from 0.3 to 424 µg Si g^−1^ followed by lungs (concentrations up to 54 µg Si g^−1^). After repeated oral exposure, Si concentrations up to 2 µg Si/g tissue were measured in the main target tissues at day six. The method developed in this study allowed the detection of Si in >88% of the analyzed samples.

### 3.5. Analytical Quality Control

In the toxicokinetic studies [22] analytical quality control of total Si measurements was accomplished using a Shewhart2 control chart (CC) [28]. The central line consisted of the QCM target value, whereas the limits were set on the basis of the SD_pooled_. In particular, the warning limits and the action limits were placed at a distance of 2 × SD and 3 × SD, respectively, on each side of the central line [48]. The data were tested for normality by the Ryan–Joiner test and no departure from normality was observed (*p* > 0.1; R − J = 0.983; α = 0.05).

The quality check of analyses was carried out taking into account the main Westgard rules [49,50,51,52]. No points were found out of control and no trend was highlighted. As a consequence, the whole process was under statistical control and reliable analytical results were produced in the course of the study.

**Figure 4:**
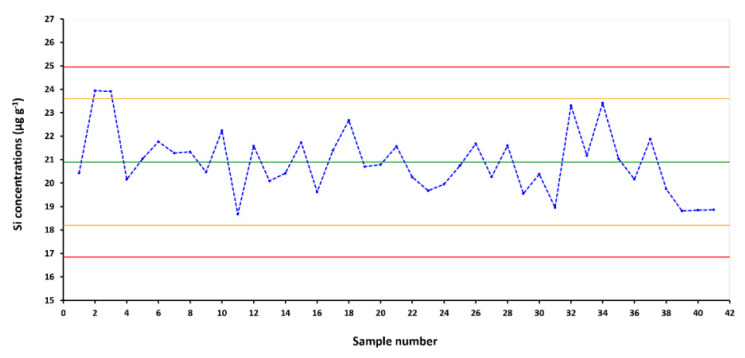
Control chart obtained over 6 months of measures for quality control material (QCM) by ICP-DRC-MS.

The complete CC, showing all the measurements performed within the study, is shown in Figure 4, and the standard deviation reflects the reproducibility of the analytical method over 6 months.

### 3.6. Screening the Presence of Particulate SiO_2_ in Tissues by spICP-MS

Single particle ICP-MS was applied to investigate the nature (particulate versus ionic) of the silicon deposited in liver of NM-203 IV-treated animals.

Being based on atomic mass spectrometry, spICP-MS is an element-specific counting technique (i.e., providing information on the chemical identity) and has the potential to measure the size, size distribution, number and mass concentration of particles. Briefly, spICP-MS is based on time resolved analysis of diluted nanoparticle dispersions using short dwell times (≤10 ms). Each particle gives rise to a signal clearly distinguishable from random background noise and, by means of appropriate algorithms and assumptions (i.e., spherical shape), signal frequency distributions are converted into size frequency distributions [53,54,55,56,57].

**Figure 5:**
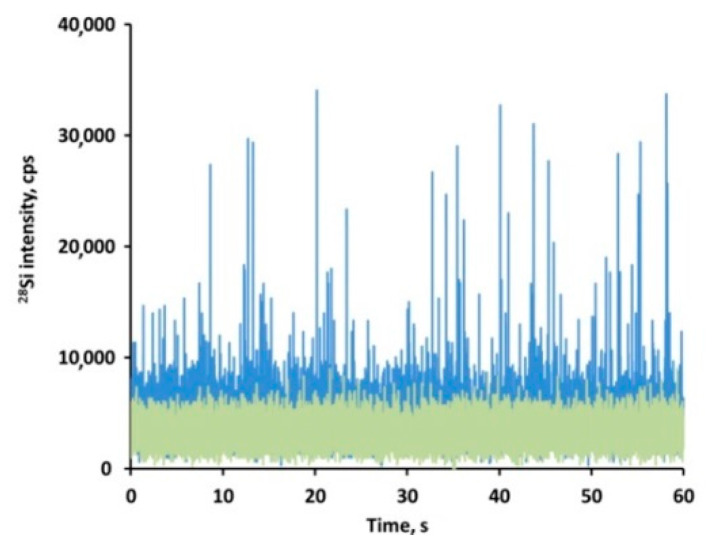
Time scan of control (green) and NM-203 treated (blue) liver samples in spICP-MS.

Liver sample extracts were analyzed after enzymatic digestion, which was applied to liberate the particles from the matrix. The concentration LoD was 0.02 µg g^−1^ whereas the size LoD was 350 nm, both calculated on procedural blanks according to Yongbo et al. [58]. Single-particle ICP-MS revealed that control and treated animals showed a clearly different pattern (Figure 5). Agglomerates of SiO_2_ nanoparticles in the size range 350–900 nm were consistently found in liver of animals belonging to the SAS-treated group. This demonstrated deposition of silica particles after short-term (5 days) IV exposure to SAS. Whether primary SiO_2_ nanoparticles formed agglomerates in vivo or the detected agglomerates are an artefact of sample preparation or spICP-MS detection cannot be unambiguously established.

**Figure 6:**
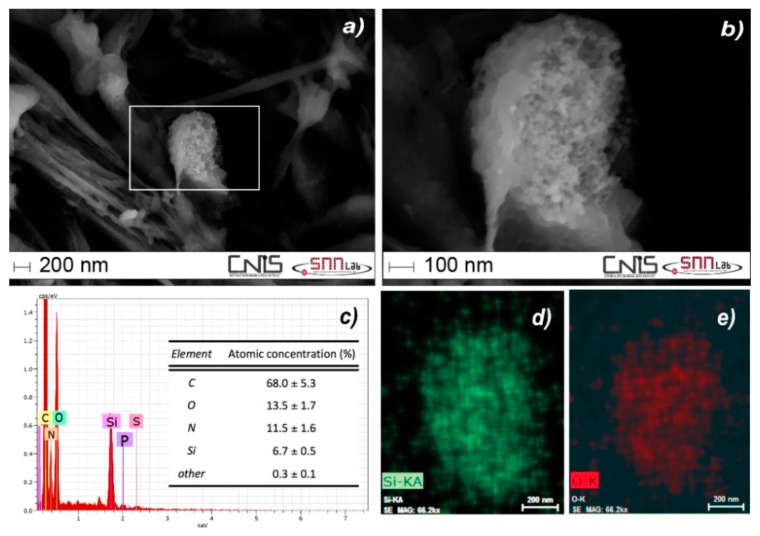
SEM micrograph of a portion of liver tissue from a NM-203 IV-treated animal showing a silica agglomerate at a magnification of 65.00 k× (**a**) and a magnification of 200.00 k× (**b**); (**c**) portion of the EDX spectrum documenting the presence of Si and O, from which the agglomerate is identified as SiO_2_; compositional EDX maps of Si (**d**) and O (**e**).

The presence of SiO_2_ agglomerates in liver tissue was confirmed by scanning electron microscopy with energy dispersive X-ray spectrometry (Figure 6). SEM-EDX identified secondary particles in the size range 200–2200 nm, where the lower bound value corresponds to the size LoD of the technique in the biological matrix.

On average, particulate SiO_2_ detected by spICP-MS accounted for about 10% of the total-Si concentration, but the true particulate fraction of total Si present in liver tissue was certainly larger since particles smaller than 350 nm were detected by SEM-EDX and these particles escaped spICP-MS detection because their diameter was below the size LoD. In addition, it is likely that agglomerates smaller than 200 nm and primary particles were also present, but could not be detected via either SEM-EDX or spICP-MS.

## 4. Discussion

ICP-MS-based analytical platforms present several advantages for ADME studies of inorganic nanomaterials. Biodistribution of particles can be tracked effectively via the concentration of their characteristic chemical element, since the detection power of the technique enables low-level analysis. The wide linear dynamic range and high sample throughput are additional desirable features, which prove particularly advantageous when a number of samples with widely variable concentrations have to be analyzed. In addition, the same platform can be operated in single particle mode to selectively detect the particles containing the chemical element of interest.

In earlier studies we demonstrated the merits of ICP-MS based analytical platforms in investigating agglomeration and dissolution of silica nanoparticles in aqueous suspensions [4] and in their quantitative characterization by means of hyphenated methods based on on-line coupling with fractionation techniques [19]. The present study focused on the analytical challenges related to the determination of silicon and silica particles in biological samples, specifically rat tissues (blood and organs) and rodent diets, with a view to developing a comprehensive strategy for SAS toxicokinetic studies in rodents.

As far as determination of silicon is concerned, high-throughput sample treatment tailored to tissue type and based on a microwave dissolution method capable of converting silica particles into soluble silicon was developed. To that aim, HF had to be added to the acid mixture but the selected HF concentrations prevented precipitation of fluorides and the need to add boric acid. An entirely novel component of the sample preparation approach developed was the vessel decontamination study, which enabled a drastically reduced silicon carry-over and a decreased magnitude and variability of silicon levels in MW irradiated method blanks. This was instrumental to the obtainment of LoD and LoQ values 1–2 orders of magnitude lower than to those achieved in other in vivo studies with SiO_2_-based materials [34,35,36,37], thus allowing the administration of low, biologically relevant doses of the SAS test materials in the toxicokinetic studies for which the analytical method presented herein was developed [22].

The effective removal of spectral interferences affecting the analytical mass was another key component of the method developed for total silicon determination. We previously demonstrated chemical resolution of polyatomic interferences when silica particles in aqueous suspensions are analyzed [4,19]. However, when biological matrixes are addressed the magnitude of such interferences (especially from ^14^N^14^N^+^ and ^12^C^16^O^+^) is substantial due to the residual C and N present in digested samples. By using methane as reaction gas, conditions for interference-free detection of total silicon were identified.

The paucity of biological reference materials available to check the accuracy of silicon measurements was another obstacle addressed in the present study. We prepared a biological QCM and characterized its silicon content by three independent techniques (ICP-DRC-MS, ICP-OES, and HR-ICP-MS). The QCM was a central component of the comprehensive quality control strategy we successfully developed to obtain reliable analytical results.

One of the main challenges in undertaking toxicokinetic studies of SAS materials via silicon determination is the high endogenous silicon background in tissues and biological fluids. For the first time, we showed that minimization of silicon background in rat tissues can be achieved by feeding animals a non-high Si diet. We investigated the variability in silicon content of available standard rat diets and selected the one with the lowest amount of silicon for use in the SAS toxicokinetic studies [22]. As a result, silicon background was reduced below the analytical LoD in circulating blood and below the analytical LoQ in most organs.

Another key issue addressed by the present study for the first time was the investigation of the nature (particulate versus ionic) of the silicon deposited in target organs of SAS-treated animals. An approach for the extraction of intact particles from biological tissues was required for such an investigation. Enzymatic digestion was applied to liberate potentially present particles from the matrix and their presence (as agglomerates) was demonstrated in treated as opposed to untreated (control) animals. SAS particles were thus showed to be (at least partly) deposited as such in target organs.

## 5. Conclusions

An analytical strategy for performing SAS toxicokinetic studies in rodents using an ICP-MS based analytical platform was developed. Minimization of silicon background in tissues was achieved by selecting a standard rodent diet low in Si. Tissue levels were measured as Si via microwave dissolution with mixtures of HNO_3_, H_2_O_2_, and HF followed by ICP-MS detection with chemical resolution of spectral interferences. As far as microwave digestion is concerned, a cleaning procedure to reduce Si carry-over was developed and sample treatment was tailored to tissue type so as to ensure high-throughput. For ICP-MS determination, quartz was completely removed by the sample introduction system of the mass spectrometer and methane was used as reaction gas to eliminate polyatomic interferences. Si contamination during sample preparation was prevented by using clean room conditions, avoiding use of glass or quartz labware, and resorting to ultrapure reagents only. Two commercial SAS materials used in the food industry, one precipitated (NM-200) and one pyrogenic (NM-203), were used for method development. The whole analytical method was validated and LoDs in the range 0.2–0.5 µg Si g^−1^ for most tissues were obtained, highlighting that the method is fit for the purpose of performing ADME studies of nanosilica.

A comprehensive analytical quality control strategy was concurrently developed. Due to the paucity of biological reference materials available to check the quality of Si determination, an internal QCM was prepared and characterized by three independent techniques (ICP-DRC-MS, ICP-OES, and HR-ICP-MS). As the method was applied to toxicokinetic studies of NM-200 and NM-2003, the QCM was included in each analytical batch along with other QC samples and a Shewhart2 CC documented the obtainment of reliable analytical results in the course of the study.

Finally, single particle ICP-MS was applied to investigate the nature (particulate versus ionic) of the silicon deposited in organs after SAS administration. SiO_2_ agglomerates in liver tissue were detected and their presence was confirmed by SEM-EDX analysis.

## Figures and Tables

**Table 1:** Optimized instrument parameter.

Instrument Parameter	Value
RF Power	1.3 kV
Plasma Gas Flow	15.5 l min^−1^
Aux Gas Flow	1.2 l min^−1^
Carrier gas	1.00 l min^−1^
DRC	CH_4_
	Cell gas flow 0.8 mL min^−1^
	RPq 0.55
	CPV −17 V
	QRO −6 V
	CRO −8 V
Internal standard	^74^Ge
Analytical mass	^28^Si
Sample flow rate	1 mL min^−1^
Sweeps	20
Dwell time	200 ms
Reading	1
Replicates	3
Integration time	4 s

**Table 2:** Si determination by ICP-DRC-MS: limits of detection (LoDs) and limits of quantification (LoQs) for the different organs and blood (µg Si g^−1^ fresh weight).

Tissue	LoD	LoQ
Liver	0.3	0.9
Spleen	0.4	1.5
Lungs	0.5	1.8
Heart	0.4	1.2
Brain	0.2	0.8
Kidneys	0.3	0.9
Ovaries	1.8	6.1
Testis	0.2	0.8
GI tract	0.3	1.1
Mesenteric lymph nodes	0.4	1.5
Blood	0.2	0.7

**Table 3:** Relative recoveries expressed as percentage for NM and target organs after closed-vessel (HP) and Multiprep oxidative digestion, calculated with Equations (1) (NM) and (2) (liver and spleen).

Sample	NM-203		NM-200	
	HP	Multiprep	HP	Multiprep
NM	104.1 ± 8.4	95.5 ± 10.9	97.0 ± 2.9	97.8 ± 4.0
Liver	96.2 ± 2.2	88.1 ± 3.6	91.4 ± 2.2	86.8 ± 3.0
Spleen	86.7 ± 4.5	87.3 ± 1.7	85.0 ± 3.7	87.8 ± 3.1

**Table 4:** Total Si concentration range measured in rat tissues after IV single dose. Results are expressed as µg Si g^−1^. Measurement errors are expressed as standard deviation in brackets.

Sample	Control		NM-200		NM-203	
	Min	Max	Min	Max	Min	Max
Liver	≤0.3	0.6 (0.1)	0.9 (0.1)	116.9 (3.5)	0.4 (0.1)	114.1 (3.4)
Spleen	≤0.4	≤0.4	≤0.4	55.8 (1.7)	≤0.4	270.6 (8.1)
Lungs	≤0.5	1.1 (0.1)	≤0.5	54.3 (1.6)	≤0.5	25.0 (1.1)
Heart	≤0.4	0.8 (0.1)	≤0.4	1.9 (0.1)	≤0.4	2.6 (0.2)
Brain	≤0.2	0.7 (0.1)	≤0.2	0.5 (0.1)	≤0.2	1.5 (0.1)
Kidneys	≤0.3	0.6 (0.1)	≤0.3	1.5 (0.1)	≤0.3	1.5 (0.1)
Testis	1.0 (0.1)	1.6 (0.2)	0.8 (0.1)	1.3 (0.1)	0.8 (0.1)	1.4 (0.1)
Ovaries	≤1.8	≤1.8	≤1.8	≤1.8	≤1.8	≤1.8

**Table 5:** Total Si concentration range measured in rat tissues after IV repeated dose. Results are expressed as µg Si g^−1^. Measurements error are expressed as standard deviation in brackets.

Sample	Control		NM-200		NM-203	
	Min	Max	Min	Max	Min	Max
Liver	≤0.3	0.5 (0.1)	12.2 (0.9)	419.8 (12.6)	7.8 (0.5)	283.0 (8.5)
Spleen	≤0.4	≤0.4	12.2 (0.9)	227.1 (6.8)	17.9 (1.3)	424.0 (12.7)
Lungs	≤0.5	1.2 (0.1)	2.9 (0.2)	101.5 (3.0)	1.4 (0.1)	82.5 (3.7)
Heart	≤0.4	0.8 (0.1)	≤0.4	4.0 (0.3)	≤0.4	9.0 (0.6)
Brain	≤0.2	0.7 (0.1)	0.3 (0.1)	0.7 (0.1)	0.3	0.9 (0.1)
Kidneys	≤0.3	0.6 (0.1)	0.4 (0.1)	3.2 (0.2)	≤0.3	7.6 (0.5)
Testis	1.0 (0.1)	1.6 (0.1)	0.7 (0.1)	1.4 (0.1)	0.6 (0.1)	2.3 (0.2)
Ovaries	≤1.8	≤1.8	≤1.8	≤1.8	≤1.8	≤1.8

## Supplementary Materials

The following are available online at https://www.mdpi.com/2079-4991/10/5/888/s1, Figure S1: HR-ICP-MS mass spectrum, Figure S2: Optimization profiles of gas flow rate in ICP-DRC-MS, Table S1: Test materials, Table S2: Spectral interferences affecting ICP-MS determination of silicon, Table S3: HR-ICP-MS operating conditions, Table S4: ICP-OES operating conditions.
