# Supplementary material for: Determination of Total Silicon and SiO2 Particles Using an ICP-MS Based Analytical Platform for Toxicokinetic Studies of Synthetic Amorphous Silica
Source: Nanomaterials (Basel). 2020 May 6;10(5):888. doi: 10.3390/nano10050888 (PMC7279390; doi:10.3390/nano10050888)
Supplement: Supplementary file 1 [file nanomaterials-10-00888-s001.pdf]

nanomaterials

MDPI

Supplementary Material

Determination of Total Silicon and SiO_2 Particles Using an ICP-MS Based Analytical Platform for Toxicokinetic Studies of Synthetic Amorphous Silica

Federica Aureli [1], Maria Ciprotti [1], Marilena D'Amato [1], Emanueli do Nascimento da Silva [2,3], Stefano Nisi [4], Daniele Passeri [5,6], Angela Sorbo [1], Andrea Raggi [1], Marco Rossi [5,6] and Francesco Cubadda [1,*]

1 Istituto Superiore di Sanità-National Institute of Health, Rome 00161, Italy; federica.aureli@iss.it (F.A.); maria.ciprotti@iss.it (M.C.); marilena.damato@iss.it (M.D.); angela.sorbo@iss.it (A.S.); andrea.raggi@iss.it (A.R.)
2 Department of Chemistry, Institute of Exact and Biological Sciences, Federal University of Ouro Preto, Ouro Preto 35400000, MG, Brazil; emanueli.silva@ufop.edu.br
3 Institute of Chemistry, University of Campinas, Campinas, 13083970, SP, Brazil
4 Gran Sasso National Laboratory, National Institute of Nuclear Physics (LNGS-INFN), Assergi (AQ), 67100, Italy; stefano.nisi@lngs.infn.it
5 Department of Basic and Applied Sciences for Engineering, University of Rome Sapienza, Rome, 00161, Italy; daniele.passeri@uniroma1.it (D.P.); marco.rossi@uniroma1.it (M.R.)
6 Research Center for Nanotechnology Applied to Engineering of Sapienza University of Rome (CNIS), University of Rome Sapienza, Rome, 00185, Italy
* Correspondence: francesco.cubadda@iss.it; Tel.: +39-0649906024

Received: 31 March 2020; Accepted: 28 April 2020; Published: date

Test Materials

Table S1. Main physical and chemical characteristics of SAS NM used in this study.

NM-code	Primary particle size[1] (nm)	Particle size distribution (nm)	Purity[2] (wt %)	Main impurities[2] (mg g^{-1})	BET SSA[3] (m^2/g)
NM-200	14-23	<100: 89%<br><50: 70%<br><10: 2%	96.5	Na (8.8)<br>S (4.6)<br>Al (8.7)	189.2
NM-203	13-45	<100 nm: 77 %,<br><50 nm: 48%<br><10 nm: 0.3%	99.3	Na (ND[4])<br>S (0.4)<br>Al (4.3)	203.9

[1] Determined by transmission electron microscopy
[2] Determined by energy dispersive spectrometry
[3] BET (Brunauer-Emmet-Teller) specific surface area (SSA). Determined by nitrogen adsorption
[4] ND Not detected

Spectral interferences affecting ICP-MS determination of silicon

Table S2. Silicon naturally occurring isotopes and polyatomic interferences in ICP-MS.

Isotope	Abundance of	Interfering species

	**isotope**	
^{28}Si	92.21	$^{14}N^{14}N^+$, $^{12}C^{16}O^+$
^{29}Si	4.7	$^{14}N^{15}N^+$, $^{14}N^{14}NH^+$, $^{13}C^{16}O^+$, $^{12}C^{16}OH^+$
^{30}Si	3.09	$^{15}N^{15}N^+$, $^{14}N^{15}NH^+$, $^{14}N^{16}O^+$, $^{13}C^{17}O^+$, $^{12}C^{17}OH^+$

HR-ICP-MS analysis

The measurements were carried out using an ELEMENT2 High Resolution Inductively Coupled Plasma Mass Spectrometer (HR-ICP-MS) from Thermo Fischer Scientific. The instrument, installed in a clean room ISO 5, was equipped with an ASX520 autosampler from CETAC and an impact bead Spray Chamber. The instrumental conditions adopted for the analysis are shown in Table S3. With the conditions used in this study, ^{28}Si and ^{29}Si peaks are completely resolved from interfering ions at medium resolution (Figure S1).

Table S3. Instrumental parameters used for HR-ICP-MS analysis.

Instrument Parameter	Value
RF Power	1.29 kW
Reflected power	<5 W
Shielding	Guard Electrode System
Run/pass	3/9
Sample time	10 ms
Sample per peak	20
Integration window	80% of theoretical peak
Analytics	^{28}Si, ^{29}Si, ^{72}Ge, MR

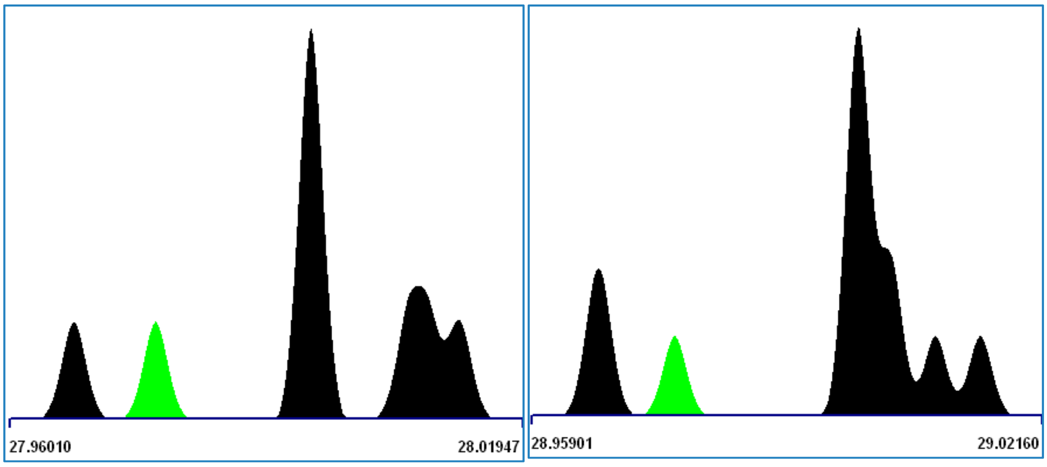

Figure S1. Mass spectrum of the ^{28}Si (left panel) and ^{29}Si (right panel) for the QC-ISS QCM as measured with the ELEMENT2 HR-ICP-MS in medium resolution mode (M/ΔM @4000). Analytical masses in green, interfering ions in black.

ICP-OES analysis

The measurements were carried out using a Perkin Elmer 4300DV High Resolution Inductively Coupled Plasma Optical Emission Spectrometer (ICP-OES) (Perkin Elmer, Norwalk, CT) equipped with a ceramic torch and a sapphire injector. The instrumental conditions adopted for the analysis are shown in Table S4.

Table S4. Instrumental parameters used for ICP-OES analysis.

Instrument Parameter	Value
RF Power	1.4 kW
Spray Chamber	Cyclonic
Nebulizer	Concentric
Carrier gas (Ar) flow rate	15 L min^{-1}
Auxiliary gas (Ar) flow rate	0.2 L min^{-1}
Coolant gas (Ar) flow rate	0.215 L min^{-1}
Nebulizer flow	0.75 mL min^{-1}
Observation height (mm)	15
Integration time	5
Wavelength	Si (251.611); Y as internal standard (371.029)

ICP-MS analysis: chemical resolution of interferences

The reaction profiles obtained when Methane, Oxygen, Hydrogen and Ammonia were investigated as reaction gases are shown in Figure S2.

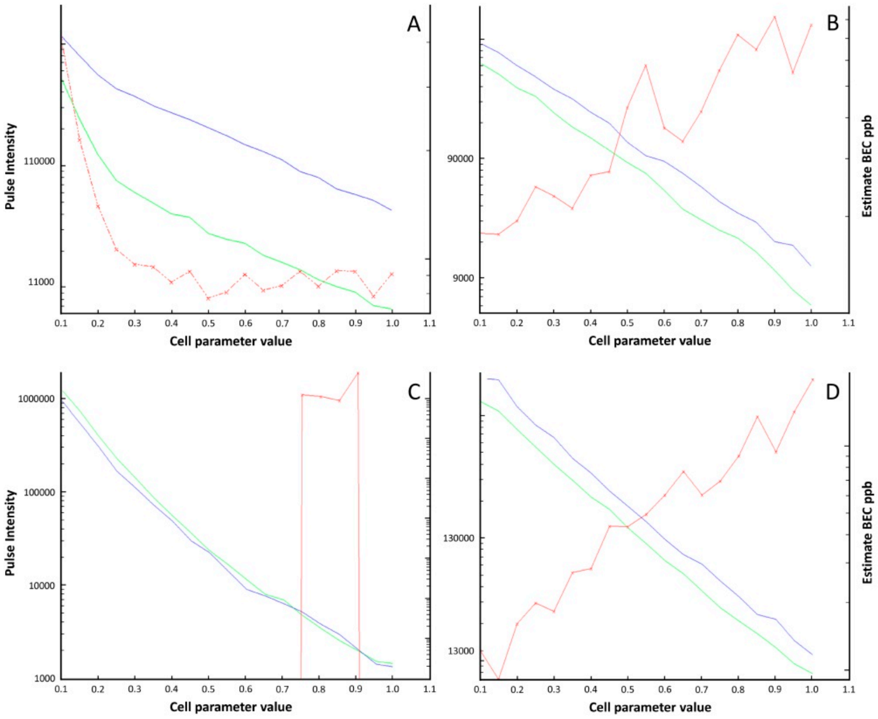

Figure S2. Optimization profiles of gas flow rate in a liver sample with methane (A), Oxygen (B), Hydrogen (C) and ammonia (D). The blue line corresponds to the analyte signal (matrix spiked with 250 μg Si/L) and the green line to the background signal (matrix). The red line represents the BEC.
